# Association of modified NUTRIC score for nutritional risk and in-hospital developed malnutrition in adults with severe injuries: a prospective observational cohort study

**DOI:** 10.1007/s00068-025-02887-7

**Published:** 2025-05-20

**Authors:** Esmee A. H. Verheul, Dylan Koole, Suzan Dijkink, Pieta Krijnen, Jochem M. Hoogendoorn, Sesmu Arbous, Ron Peters, George C. Velmahos, Ali Salim, Daniel D. Yeh, Inger B. Schipper

**Affiliations:** 1 Department of Trauma Surgery, Leiden University Medical Center, Post zone K6-R, P.O. Box 9600, Leiden, 2300 RC The Netherlands; 2 Department of General Surgery, Haaglanden Medical Center, The Hague, The Netherlands; 3 Acute Care Network West Netherlands, Leiden, the Netherlands; 4 Department of Intensive Care, Leiden University Medical Center, Leiden, The Netherlands; 5 Department of Intensive Care, Haaglanden Medical Center, The Hague, The Netherlands; 6 Department of Trauma Surgery, Massachusetts General Hospital, Boston, MA USA; 7 Department of Surgery, Brigham and Women’s Hospital, Boston, MA USA; 8 Department of Surgery, Denver Health Medical Center, Denver, CO USA

**Keywords:** Nutritional risk, Trauma, Complications, Severely injured, Intensive care unit

## Abstract

**Background:**

This study investigated the prevalence of high nutritional risk (modified Nutrition Risk in Critically Ill (mNUTRIC) score ≥ 5) and its relation with malnutrition and other adverse in-hospital outcomes in severely injured patients (Injury Severity Score ≥ 16), admitted to the ICU. We hypothesized that high nutritional risk is associated with an increased risk of developing malnutrition (primary hypothesis) and of complications and mortality (secondary hypotheses) in adults with severe injuries compared to those with low nutrition risk.

**Methods:**

In this observational prospective study, 100 severely injured patients admitted to the ICU of five Level-1 trauma centers in the US and the Netherlands between 2018–2022 were included. During ICU and hospital stay, malnutrition rates (Subjective Global Assessment score ≤ 5), complication rates (systemic complications, pneumonia, urinary tract infection, venous thromboembolism), and mortality of severely injured patients with high versus low nutritional risk were compared. A cause-specific Cox regression model was fitted to analyze whether high nutritional risk was related to developing malnutrition.

**Results:**

Eighteen percent of patients had high nutritional risk (95% confidence interval [CI] 10.5–25.5%) at admission. High nutritional risk was not related to in-ICU or in-hospital developed malnutrition. In patients with high nutritional risk, the hazard ratio for developing malnutrition was 1.3 (95% CI 0.7–2.6, *p* = 0.45). Severely injured patients with high nutritional risk had more complications during ICU (78% vs 29%, *p* < 0.001; OR 8.5, 95% CI 2.5–28.3) and hospital stay (83% vs 41%, *p* < 0.01; OR 6.0, 95% CI 1.5–24.9). ICU mortality (22% vs 4%, *p* = 0.02; OR 7.5, 95% CI 1.5–37.3) and hospital mortality (33% vs 6%, *p* < 0.01; OR 5.9, 95% CI 1.3–26.4) were also higher in patients with high nutritional risk.

**Conclusion:**

About one-fifth of severely injured patients admitted to the ICU had high nutritional risk. High nutritional risk in severely injured patients is not associated with malnutrition. It is potentially associated with adverse in-hospital outcomes.

**Level of evidence:**

Level III, Prognostic/Epidemiological.

**Supplementary Information:**

The online version contains supplementary material available at 10.1007/s00068-025-02887-7.

## Introduction

Malnutrition is reported to be independently associated with higher mortality risk, longer hospital length of stay (LOS), and increased cost of hospitalization [1, 2]. A variety of tools are available to assess the nutritional status, including nutritional screening tools to assess the risk of developing malnutrition and nutritional assessment tools to evaluate current nutritional status and diagnose malnutrition [3]. Assessing malnutrition using a nutritional assessment tool remains a significant challenge in severely injured patients, as obtaining their dietary history is often complicated by decreased consciousness and/or the need for mechanical ventilation. Evaluation of muscle wasting can be misleading due to swelling and edema, and serum levels of visceral proteins (albumin and pre-albumin) concentrations are affected by the acute-phase response after inflammation or trauma [4–6].

Alternatively, nutritional screening tools can assess the risk of developing malnutrition and enable timely initiation of appropriate nutritional interventions. This proactive approach helps prevent the onset and progression of malnutrition, along with its associated complications. Among the nutritional screening tools, the modified Nutrition Risk in the Critically Ill (mNUTRIC) score is a validated tool used to quantify the risk of malnutrition and adverse outcomes that may be modified by nutrition therapy in the critical care setting [7]. The mNUTRIC score is based on age, Acute Physiology and Chronic Health Evaluation II (APACHE II) score, Sequential Organ Failure Assessment (SOFA) score, number of comorbidities, and days in-hospital prior to ICU admission [8]. The prevalence of high nutritional risk, defined as mNUTRIC score ≥ 5, ranges from 22 to 91% in critically ill patients [9].

Little is known about the nutritional risk of severely injured patients. Timely identification of patients at risk for malnutrition is essential as severely injured patients experience a hypermetabolic state after severe trauma, leading to increased muscle protein mobilization for energy, and decreased protein synthesis leading to catabolism [10]. This hypermetabolic state makes them more vulnerable to acute disease-related or injury-related malnutrition involving a marked inflammatory response [11]. Consequently, an objective measure for assessing nutritional risk, such as the mNUTRIC score, can demonstrate its value if patients identified as having high nutritional risk are more likely to develop malnutrition during their admission. Then, nutritional interventions might be initiated to prevent the onset of malnutrition.

The primary goal of this observational prospective cohort study is to test the hypothesis that high nutritional risk is associated with an increased risk of developing malnutrition in adults with severely injured compared to those with low nutritional risk. Furthermore, the relation between high nutritional risk and other adverse in-hospital outcomes, including complications and mortality, in severely injured patients admitted to the ICU was assessed.

## Material and methods

### Design and setting

The Malnutrition in Polytrauma Patients (MaPP) study is an observational prospective cohort study that was performed on 100 adult severely injured patients at five Level-1 trauma centers, three in the United States (Massachusetts General Hospital and Brigham and Women’s Hospital at Boston, and Ryder Trauma Center in Miami) and two in the Netherlands (Leiden University Medical Center at Leiden and Haaglanden Medical Center Westeinde at The Hague). The study was conducted according to the guidelines of the Declaration of Helsinki and approved by the local Institutional Review Boards (protocol number Netherlands: NL64016.058.17, approved on February 21, 2018; protocol number USA: 2018P000202/PHS, approved on April 3, 2018). This study is reported in line with the Strengthening the Reporting of Observational Studies in Epidemiology (STROBE) Statement [12]. The study methods are described in detail in the published study protocol [13].

### Inclusion and exclusion criteria

All consecutive adult (≥ 18 years) patients with severe injuries (defined as Injury Severity Score, ISS ≥ 16) caused by blunt trauma, admitted to the ICU of one of the participating centers, were eligible for inclusion. Patients needed to be admitted to the ICU for more than 48 h and were not primarily managed in another hospital. Patients with burn wounds and penetrating injuries were excluded.

### Patient enrolment

Trauma patients newly admitted to the ICU were screened for inclusion criteria upon admission by the investigators at the participating hospitals between July 2018 and April 2022. Eligible patients were asked to provide written informed consent for participation in the study. If the patient was unable to provide consent (e.g., due to unconsciousness), a legal representative was asked to provide informed consent. If a legal representative gave consent, and the patient became able to provide consent later in the study, they were asked to confirm it themselves. In cases where the patient did not have a legal representative, data was collected prospectively, and the patient was asked for consent once they could do so. If the patient declined to participate in the study, their data was removed from the electronic database. The patient and/or their legal representative could withdraw consent and exit the study at any time.

### Sample size

As described in the study protocol, the a priori sample size calculation showed that 195 patients were needed to answer the primary question of the MaPP study [13]. Due to the low inclusion rate during the COVID-19 pandemic, it was decided to prematurely end the inclusion at 100 patients.

### Study parameters

#### Nutritional risk

Our exposure of interest was high nutritional risk defined by modified Nutrition Risk in the Critically Ill (mNUTRIC) ≥ 5 [8]. Our comparator was low nutritional risk defined by mNUTRIC < 5. The mNUTRIC score was determined by trained personnel within 24 h after ICU admission. This score is based on five items: age, Acute Physiology and Chronic Health Evaluation II (APACHE II) score [14], Sequential Organ Failure Assessment (SOFA) score [15], the number of comorbidities, and number of days in-hospital prior to ICU admission. The APACHE II score measures ICU mortality based on a number of laboratory values and patient signs. The SOFA score uses measurements of major organ function to determine the degree of organ failure. The mNUTRIC, APACHE II, and SOFA scores are listed in the Appendix.

#### In-hospital outcomes

The primary outcome was malnutrition, defined as a Subjective Global Assessment (SGA) score ≤ 5. The SGA score was assessed at ICU admission, every five days during ICU stay, at ICU discharge, weekly during admission to the ward, and at hospital discharge [16]. The SGA is a nutritional assessment tool that has been validated for the acute hospital setting, surgical patients, and patients admitted to the ICU requiring mechanical ventilation [17–19]. The SGA score is shown in the Appendix.

Secondary outcomes were complications, including systemic complications (sepsis, Acute Respiratory Distress Syndrome (ARDS), Systemic Inflammatory Response Syndrome (SIRS), multiple-organ failure), pneumonia, urinary tract infection (UTI), deep venous thrombosis (DVT), and pulmonary embolism (PE). Mortality was analyzed as a separate outcome parameter. This was also described in the study protocol [13].

Patient demographics, including age, sex, and body mass index (BMI), were recorded, along with trauma characteristics such as the Abbreviated Injury Scale (AIS) for all body regions and the ISS. Information on nutritional support was collected, and patients were categorized based on whether they received oral feeding or (par)enteral feeding. For the patients who received (par)enteral nutrition, the timing of its administration was documented, and whether it was initiated within 48 h or after 48 h of admission. Target energy goals were calculated through a weight-based predictive Eq. (25 kcal/kg/day). In overweight patients (BMI > 25 kg/m^2^), the adjusted body weight was used, which is calculated through the ideal body weight. The ideal body weight is calculated by the following equation: 0.9 × height in cm—100 (male) (or—106 (female)). To account for the metabolic demand of adipose tissue and muscle, an additional 25% of the excess weight (actual body weight minus ideal body weight) is added to the ideal body weight to calculate the adjusted weight [20]. According to the ESPEN guidelines, target energy goals should be met after 3–7 days of admission. It was documented whether goals were met after < 48 h, 3–7 days, and after > 7 days of admission. Surgical procedures that required patients to go to the operating room were documented. Other in-hospital outcomes included hospital length of stay (LOS), ICU LOS, and ventilator days.

### Statistical analysis

All analyses were performed using IBM SPSS Statistics for Windows, version 25 (IBM Corp., Armonk, N.Y., USA) and R version 4.2.2. *P*-values < 0.05 were considered statistically significant. The baseline characteristics of the patients with low and high nutritional risk were compared using the Chi-square test or Fisher’s exact test (in case of expected cell counts < 5) for categorical variables, the independent samples T-test for normally distributed continuous variables, and the Mann–Whitney U test for skewed continuous variables.

The prevalence of high nutritional risk was calculated as the proportion with a 95% confidence interval (CI) of patients with a mNUTRIC score ≥ 5. The malnutrition rate was calculated as the proportion of patients well-nourished at admission who developed malnutrition during admission as diagnosed with the SGA. The patients who were already malnourished at admission were excluded from this analysis. The incidences of malnutrition, complications, and mortality during ICU and total hospital stay were compared between the patients with high and low nutritional risk using the Chi-square test. Furthermore, a cause-specific Cox regression model was fitted to analyze whether high nutritional risk was related to developing malnutrition during hospital admission. In this model, receiving (par)enteral feeding was added as a binary time-dependent covariate.

## Results

### Patient and trauma characteristics

The median age of the 100 included severely injured patients was 51 (interquartile range (IQR) 32–64) years, and 70 patients were male (Table 1). 59 patients were involved in motor vehicle accidents, 37 fell from a height, and 4 sustained injuries from other causes. Severe head trauma (AIS ≥ 4) was the most common, and 67% were considered to be very severely injured (ISS ≥ 25). 4 patients underwent abdominal surgery, 1 in the high nutritional risk and 3 in the low nutritional risk group. 52 patients had a healthy weight (BMI 18.5–25 kg/m^2^), 30 were overweight (BMI 25–30 kg/m^2^), and 19 were classified as obese (BMI ≥ 30 kg/m^2^). Eighteen patients were considered to have high nutritional risk (mNUTRIC ≥ 5) at admission (18%, 95% CI 10.5–25.5%). As expected, patients with a high and low nutritional risk differed with respect to the five mNUTRIC score items (Table 1). Patients with high nutritional risk were more frequently obese (44% vs 13%; *p* = 0.02). Twelve patients were malnourished at admission, 17% in the high nutritional risk group vs 11% in the low nutritional risk group (*p* = 0.45).

**Table 1:** Patient characteristics according to nutritional risk at admission

	Total (*n* = 100)	Low nutritional risk (mNUTRIC < 5) (*n* = 82)	High nutritional risk (mNUTRIC ≥ 5) (*n* = 18)	*P* value
mNUTRIC score items				
Age in years, median (IQR)	51 (32–64)	45 (28–62)	66 (60–77)	** < 0.001**
APACHE II, median (IQR)	16 (11–20)	14 (10–18)	22 (20–27)	** < 0.001**
SOFA, median (IQR)	6 (4–8)	6 (4–8)	10 (7–11)	** < 0.001**
> 1 comorbidity*	45 (45%)	28 (34%)	17 (94%)	** < 0.001**
≥ 1 day in hospital prior to ICU admission	0 (0%)	0 (0%)	0 (0%)	**-**
Other parameters				
Male sex	70 (70%)	55 (67%)	15 (83%)	0.28
BMI category				** < 0.01**
* Healthy weight (*< *25.0)*	52 (52%)	47 (57%)	5 (28%)	
* Overweight (*≥ *25.0—*< *30.0)*	30 (30%)	24 (29%)	5 (28%)	
* Obese (*≥ *30.0)*	19 (19%)	11 (13%)	8 (44%)	
Severe injury (AIS ≥ 4)				
* Head*	44 (44%)	34 (42%)	10 (56%)	0.41
* Chest*	29 (29%)	24 (29%)	5 (28%)	1.00
* Abdomen*	9 (9%)	8 (10%)	1 (6%)	0.91
* Extremity*	14 (14%)	11 (13%)	3 (17%)	1.00
ISS ≥ 25	67 (67%)	54 (66%)	13 (72%)	0.81
Malnourished at admission (SGA ≤ 5)	12 (12%)	9 (11%)	3 (17%)	0.45
Type of nutrition				**0.02**
* Oral*	29 (29%)	28 (34%)	1 (6%)	
* (Par)enteral*	71 (71%)	54 (66%)	17 (94%)	
Initiation of (par)enteral nutrition				**0.02**
< *48 h*	63 (89%)	51 (94%)	12 (71%)	
≥ *48 h*	8 (11%)	3 (6%)	5 (29%)	
Time until target energy goals were met				0.46
< *48 h*	19 (19%)	16 (20%)	3 (17%)	
* 3–7 days*	67 (67%)	53 (65%)	14 (78%)	
> *7 days*	14 (14%)	13 (16%)	1 (6%)	
Surgery	82 (82%)	65 (79%)	17 (94%)	0.18

n(%) unless stated otherwise

*AIS* Abbreviated Injury Scale severity (last digit of the AIS code), *BMI* Body Mass Index, *ICU* Intensive care unit, *IQR* Interquartile range, *ISS* Injury Severity Score, *SD* Standard deviation, *SGA* Subjective Global Assessment

*According to the mNUTRIC comorbidity list

### Nutritional support

In patients with high nutritional risk, (par)enteral feeding was initiated more frequently than in patients with low nutritional risk (94% vs 66%; *p* = 0.02, Table 1). Furthermore, in the patients who received (par)enteral feeding, this was not initiated within the recommended 48 h in 29% of the patients with high nutritional risk, compared to 6% of the patients with low nutritional risk (*p* = 0.02). The timing until target energy goals were met did not differ statistically significant between the two groups. Additionally, among patients with high nutritional risk who did receive (par)enteral feeding, initiation within the recommended 48 h was less common compared to the patients with low nutritional risk (71% vs 94%, *p* = 0.02, Table 1).

### High nutritional risk and incidence of malnutrition

**Fig. 1:**
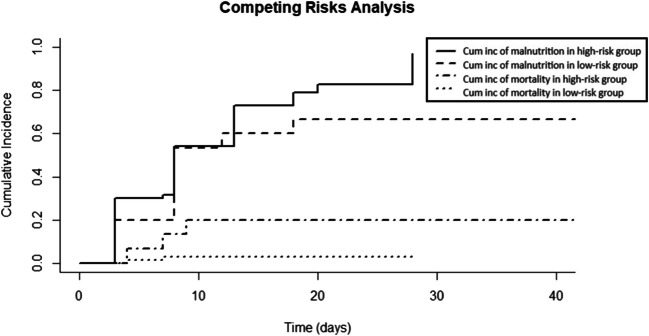
Cumulative incidence functions for malnutrition and mortality during hospital admission, stratified by nutritional risk. Cum inc, Cumulative incidence; High risk, High nutritional risk (mNUTRIC ≥ 5); Low risk, Low nutritional risk (mNUTRIC < 5); Malnutrition, Malnutrition developed during hospital admission (SGA ≤ 5); Mortality, In-hospital mortality

Of the 73 patients with low nutritional risk who were well-nourished at admission, 49% developed malnutrition during ICU stay and 71% during total hospital stay, compared to 53% and 67% respectively of the 15 patients with high nutritional risk who were well-nourished at admission (*p* = 1.00 and *p* = 0.76; Tables 2 and 3). No statistically significant difference was seen between the time to development of malnutrition during ICU and hospital stay in the patients with low and high nutritional risk (Tables 2 and 3). Figure 1 shows the cumulative incidence of malnutrition and mortality during hospital stay for the patients with high and low nutritional risk in the cause-specific Cox regression analysis. High nutritional risk did not pose a statistically significant risk of developing malnutrition when correcting for receiving (par)enteral feeding (hazard ratio 1.31, 95% confidence interval 0.65–2.64; *p* = 0.45).

**Table 2:** Patient outcomes during ICU stay per nutritional risk group

	Total (*n* = 100)	Low nutritional risk (mNUTRIC < 5) (*n* = 82)	High nutritional risk (mNUTRIC ≥ 5) (*n* = 18)	*P* value
Malnutrition developed during ICU admission*	44 (50%)	36 (49%)	8 (53%)	1.00
Time to develop malnutrition (days), mean ± SD (days), mean ± SD	6.0 ± 4.6	5.8 ± 4.8	7.2 ± 3.0	0.41
Complication	38 (38%)	24 (29%)	14 (78%)	** < 0.001**
Systemic complications	10 (10%)	5 (6%)	5 (28%)	**0.02**
Pneumonia	32 (32%)	20 (24%)	12 (67%)	** < 0.01**
Urinary tract infection	4 (4%)	2 (2%)	2 (11%)	0.30
Venous thromboembolism	4 (4%)	4 (5%)	0 (0%)	0.77
ICU mortality	7 (7%)	3 (4%)	4 (22%)	**0.02**
ICU LOS, mean ± SD**	13 ± 18	12 ± 16	22 ± 26	0.16
Ventilator days, mean ± SD**	8 ± 14	6 ± 9	19 ± 27	0.12

n(%) unless stated otherwise

*ICU* Intensive care unit, *LOS* Length of stay, *n* number, *SD* Standard deviation

*Patients that were malnourished at ICU admission were excluded (*n* = 12), comprising 9 patients in the low nutritional risk group and 3 patients in the high nutritional risk group

**Patients that died during ICU admission were excluded (*n* = 7)

**Table 3:** Patient outcomes during hospital stay per nutritional risk group

	Total (*n* = 100)	Low nutritional risk (mNUTRIC < 5) (*n* = 82)	High nutritional risk (mNUTRIC ≥ 5) (*n* = 18)	*P* value
Malnutrition developed during hospital admission*	62 (70%)	52 (71%)	10 (67%)	0.76
Time to develop malnutrition	7.8 ± 5.3	7.8 ± 5.5	7.9 ± 4.6	0.97
Complication	49 (49%)	34 (41%)	15 (83%)	** < 0.01**
Systemic complications	10 (10%)	5 (6%)	5 (28%)	**0.02**
Pneumonia	40 (40%)	27 (33%)	13 (72%)	** < 0.01**
Urinary tract infection	11 (11%)	8 (10%)	3 (17%)	0.67
Venous thromboembolism	7 (7%)	6 (7%)	1 (6%)	1.00
In-hospital mortality	11 (11%)	5 (6%)	6 (33%)	** < 0.01**
Hospital LOS, mean ± SD**	29 ± 24	27 ± 24	44 ± 23	**0.03**

n(%) unless stated otherwise

*LOS* Length of stay, *n* number, *SD* Standard deviation

*Patients that were malnourished at ICU admission were excluded (*n* = 12)

**Patients that died during hospital admission (*n* = 11) or were transferred to another hospital (*n* = 2) were excluded

### High nutritional risk and other complications

Patients with high nutritional risk developed more other complications during ICU and total hospital stay than patients with low nutritional risk: 78% (*n* = 14/18) vs 29% (*n* = 24/82) during ICU stay (*p* < 0.001; Table 2) and 83% (*n* = 15/18) vs 41% (*n* = 34/82) during total hospital stay (*p* < 0.01, Table 3). In particular, pneumonia and systemic complications occurred more frequently in the patients with high nutritional risk (Tables 2 and 3).

Seven patients died during their stay at the ICU, and four more patients died while being admitted to the ward (Table 1). Twenty-two percent (*n* = 4/18) of the patients with high nutritional risk died during ICU admission compared to 4% (*n* = 3/82) of the patients with low nutritional risk (*p* = 0.02; Table 2). The in-hospital mortality was 33% (*n* = 6/18) in the patients with high nutritional risk and 6% (*n* = 5/82) in the patients with low nutritional risk (*p* < 0.01; Table 3).

Patients with high nutritional risk had a statistically significant longer hospital stay compared to those with low nutritional risk (44 ± 23 vs 27 ± 24, *p* = 0.03, Table 3).

## Discussion

The aim of this study was to investigate the prevalence of high nutritional risk and its relation with malnutrition and other adverse in-hospital outcomes in severely injured patients admitted to the ICU. Eighteen percent of these patients were considered to have high nutritional risk at admission. Nutritional risk was not related to the development of malnutrition during ICU and hospital stay. Complications, especially pneumonia and systemic complications, and mortality, occurred more often in the severely injured patients with high nutritional risk compared to the severely injured patients with low nutritional risk.

To our knowledge, no previous study has been performed on the relation between high nutritional risk and in-hospital developed malnutrition, as defined as SGA score ≤ 5. We hypothesized that patients identified as having high nutritional risk would demonstrate a correspondingly increased risk of developing malnutrition during admission. However, no relation was found between high nutritional risk and in-ICU (Table 2) and in-hospital (Table 3) developed malnutrition. In the survival analysis, patients with high nutritional risk appeared to have a higher risk of developing malnutrition (Fig. 1), but this difference was not statistically significant when the receipt of (par)enteral feeding was included in the proportional cause-specific hazard regression model.

We attempted to provide an explanation for the lack of correlation observed between the mNUTRIC and SGA score. Heyland et al. chose to select the NUTRIC variables based on comparative analyses of ICU survivors and non-survivors [7]. BMI, oral intake in the week prior to enrolment, and weight loss in the last three months were not significantly different in the survivors vs non-survivors groups, and thus not included in the mNUTRIC score. However, weight change and dietary intake are two out of six SGA items [16]. In addition, evaluating malnutrition in patients with obesity presents challenges as muscle and fat wasting are less readily apparent. Therefore, SGA-diagnosed malnutrition might be missed in patients with obesity. In our study population, the patients with high nutritional risk suffered also more frequently from obesity (Table 1). To our knowledge, the relation between the mNUTRIC score and obesity has not been stated before. However, a large meta-analysis showed that severe obesity was found to be related to increased mortality among patients experiencing blunt and/or penetrating trauma [21]. Since the mNUTRIC score is also related to mortality, this could explain the relation between obesity and the mNUTRIC score. Lastly, the goal of the mNUTRIC score is to identify patients who would benefit from aggressive nutrition intervention and the SGA score diagnoses malnutrition. Apparently, not all patients who develop SGA-diagnosed malnutrition were assumed to benefit from aggressive nutrition intervention at ICU admission according to the mNUTRIC score.

The relationship between the mNUTRIC score and malnutrition at ICU admission, as diagnosed by the SGA, has been examined in two studies involving critically ill patients [22, 23]. This finding was not confirmed in our study. Both studies concluded that high nutritional risk is not related to SGA-diagnosed malnutrition at ICU admission since these tools do not uniformly identify patients as malnourished or at high nutritional risk [22, 23]. Their explanation for not finding a correlation was that the SGA score is based on a combination of nutritional parameters prior to admission and physical status at admission, but the mNUTRIC score is largely a prospective assessment based on the expected effect of hospitalization on future nutritional status [23]. Thus, both the mNUTRIC at ICU admission and the SGA during hospital admission serve as valuable indicators for nutritional risk and nutritional status, respectively. However, attempting to find a correlation between these two tools appears to lack clinical significance.

A systematic review by Cattani et al. summarized the results of 26 studies on the prevalence of high nutritional risk using the mNUTRIC score in critically ill patients [9]. The lowest prevalence of high nutritional risk was found in a surgical ICU population by Özbilgin et al., who found a prevalence of 22.4% [24]. In a retrospective study of 771 trauma patients admitted to the ICU, the prevalence of high nutritional risk was 24.1% [25]. These percentages are comparable to our polytrauma population. In other patient groups admitted to the ICU, the prevalence of high nutritional risk ranged up to 91.1% in elderly (≥ 65 years) patients on mechanical ventilation and 88.7% in sepsis patients [26, 27].

The mNUTRIC score has extensively been researched in relation to mortality [9]. In the majority of studies, the mNUTRIC score was predictive for 28-day-, ICU-, and in-hospital mortality in critically ill patients. The association of the mNUTRIC score with adverse clinical outcomes is to be expected based on the fact that it includes disease severity–related variables such as APACHE II and SOFA, which are recognized predictors of these outcomes [14, 15]. Our study also showed a significantly higher in-hospital mortality rate in severely injured patients with high nutritional risk. In addition, we found that high nutritional risk in severely injured patients coincides with other in-hospital developed complications, such as pneumonia and sepsis. A true association cannot be established, since this analysis did not account for confounders.

## Limitations

This study is the first study to assess the relation between high nutritional risk and in-hospital developed malnutrition in severely injured patients. The sample size was limited to 100 patients for pragmatic reasons. Subsequently, the number of patients with high mNUTRIC scores was even smaller, < 20%. The small sample size may have introduced a type II error. This, and the analyses done on a high risk patient group of only 18, require careful interpretation of the results.

Not all patients who were considered eligible for the study were included. The primary reasons for this were organizational challenges as the study demanded significant time from ICU staff, and difficulties in obtaining informed consent (which can be considered burdensome for families of critically ill patients). However, we do not believe that this has led to selection bias in the included patient group, as the non-inclusion of eligible patients was at random.

We used the SGA for the assessment of the nutritional status. The SGA has been validated for ICU patients and is proven to be the most predictive for outcomes. However, the SGA is not very discriminative, since the difference between an SGA score of 5 (malnourished) or 6 (well-nourished) can be very minimal. The SGA was assessed by either a research nurse or a member of the research team, all of whom had received training in physical examination as part of their medical education and could accurately evaluate muscle mass. To enhance reliability and minimize interobserver variability, one investigator reviewed and verified all SGA scores at the conclusion of data collection. The results of the study suggest that the use of the mNUTRIC score might be valuable to identify severely injured patients at high risk of adverse in-hospital outcomes. Although the potential of mNUTRIC as an indicator for mortality and morbidity in severely injured patients seems promising, future studies with larger sample sizes and sub-analyses based on nutritional intake are needed to confirm its reliability in both trauma and non-trauma related clinical settings.

## Conclusion

About one-fifth of severely injured patients admitted to the ICU are at high nutritional risk, as assessed by the mNUTRIC score. High nutritional risk in severely injured patients does not seem to be related to malnutrition during hospital stay. It does coincide with other in-hospital developed complications and mortality in severely injured patients. In this light, the mNUTRIC score impresses as a potential indicator of morbidity and mortality in severely injured patients. Larger studies are needed to confirm these preliminary results.

## Electronic supplementary material

Below is the link to the electronic supplementary material.

Supplementary file1 (DOCX 33 KB)

## Figures and Tables

**Table 7:**
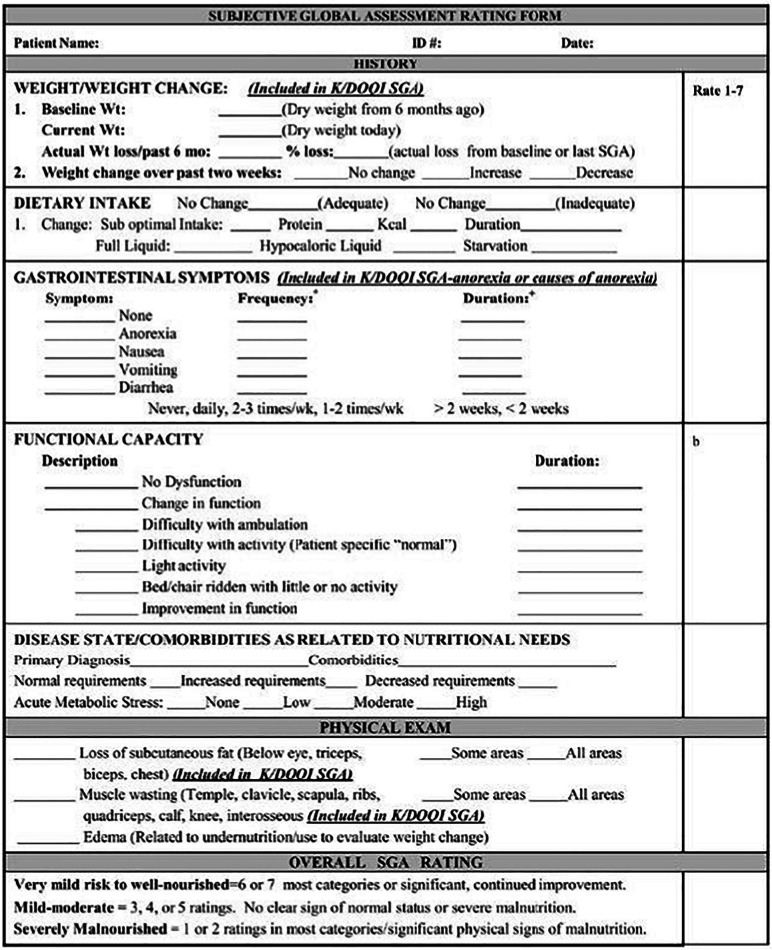
Subjective Global Assessment rating form [16]

## Data Availability

Data is provided within the manuscript.

## Appendix

see Tables 4, 5, 6 and 7

**Table 4:** Modified Nutrition Risk in Critically ill (mNUTRIC) score [8]

Variable	Range	Points
Age	< 50	0
	50–74	1
	≥ 75	2
APACHE II score	< 15	0
	15–19	1
	≥ 20	2
SOFA score	< 6	0
	6–9	1
	≥ 10	2
Number of comorbidities	0–1	0
	≥ 2	1
Days from hospital to ICU admission	0	0
	≥ 1	1

**Table 5:** APACHE II score [14]

Variables	Range	Points
Age	< 45	0
	45–54	2
	55–64	3
	65–74	5
	≥ 75	6
History of severe organ insufficiency or immunocompromised	Yes, and nonoperative or emergency postoperative patient	5
	Yes, and elective postoperative patient	2
	No	0
Rectal temperature (°C)	< 30	4
	30–32	3
	32–34	2
	34–36	1
	36–38.5	0
	38.5–39	1
	39–41	3
	≥ 41	4
MAP (mmHg)	< 50	4
	50–70	2
	70–110	0
	110–130	2
	130–160	3
	≥ 160	4
Heart rate (beats per minute)	< 40	4
	40–54	3
	55–69	2
	70–109	0
	110–139	2
	140–179	3
	≥ 180	4
Respiratory rate (breaths per minute)	< 6	4
	6–9	2
	10–11	1
	12–24	0
	25–34	1
	35–49	3
	≥ 50	4
Oxygenation (use PaO_2_ if FiO_2_ < 50%, otherwise use A-a gradient)	PaO_2_ < 55	4
	PaO_2_ 55–61	3
	PaO_2_ 61–71	1
	A-a gradient < 200 (if FiO_2_ over 49%) or pO_2_ > 70 (if FiO_2_ less than 50%)	0
	A-a gradient 200–350	2
	A-a gradient 350–500	3
	A-a gradient ≥ 500	4
Arterial pH	< 7.15	4
	7.15–7.25	3
	7.25–7.33	2
	7.33–7.50	0
	7.50–7.60	1
	7.60–7.70	3
	≥ 7.70	4
Serum sodium (mmol/L)	< 111	4
	111–119	3
	120–129	2
	130–149	0
	150–154	1
	155–159	2
	160–179	3
	≥ 180	4
Serum potassium (mmol/L)	< 2.5	4
	2.5–2.9	2
	3.0–3.4	1
	3.5–5.4	0
	5.5–5.9	1
	6.0–6.9	3
	≥ 7.0	4
Serum creatinine (mg/dL)	< 0.6	2
	0.6–1.5	0
	1.5–2.0 and chronic renal failure	2
	2.0–3.5 and chronic renal failure	3
	1.5–2.0 and acute renal failure	4
	≥ 3.5 and chronic renal failure	4
	2.0–3.5 and acute renal failure	6
	≥ 3.5 and acute renal failure	8
Haematocrit (%)	< 20	4
	20–29	2
	30–45	0
	46–49	1
	50–59	2
	≥ 60	4
White blood count (total/mm^3^)	< 1	4
	1–3	2
	3–15	0
	15–20	1
	20–40	2
	≥ 40	4
Glasgow Coma Scale	1–15	15 – Glasgow Coma Scale score

*MAP* Mean arterial pressure

**Table 6:** Sequential Organ Failure Assessment (SOFA) score [15]

Variable	Range	Points
PaO_2_/FiO_2_ (mmHg)	≥ 400	0
	< 400	1
	< 300	2
	< 200 and not MV	2
	< 200 and MV	3
	< 100 and MV	4
Platelets (× 10^3^/μL)	≥ 150	0
	100–149	1
	50–99	2
	20–49	3
	< 20	4
Glasgow Coma Scale	15	0
	13–14	1
	10–12	2
	6–9	3
	< 6	4
Bilirubin (mg/dL)	< 1.2	0
	1.2–1.9	1
	2.0–5.9	2
	6.0–11.9	3
	≥ 12.0	4
MAP (mmHg) or administration of vasoactive agents (μg/kg/min)	MAP ≥ 70	0
	MAP < 70	1
	Dopamine ≤ 5 or Dobutamine (any dose)	2
	Dopamine > 5, Epinephrine ≤ 0.1, or Norepinephrine ≤ 0.1	3
	Dopamine > 15, Epinephrine > 0.1, or Norepinephrine > 0.1	4
Creatinine (mg/dL) or urine output	< 1.2	0
	1.2–1.9	1
	2.0–3.4	2
	3.5–4.9 or urine output < 500 mL/day	3
	≥ 5.0 or urine output < 200 mL/day	4

*MAP* Mean arterial pressure, *MV* mechanically ventilated
